# Successful myomectomy during pregnancy : A case report

**DOI:** 10.1186/1742-4755-2-6

**Published:** 2005-08-16

**Authors:** Chisara Umezurike, Paul Feyi-Waboso

**Affiliations:** 1 Department of Obstetrics and Gynecology, Nigerian Christian Hospital, Aba, Nigeria; 2 Department of Obstetrics and Gynecology, Abia State University Teaching Hospital, Aba, Nigeria

## Abstract

**Background:**

The medical literature has reported an increase in myomectomy during caesarean section in the past decade. However, myomectomy performed during pregnancy remains a rarity. The management of uterine fibroids during pregnancy is usually expectant and surgical removal is generally delayed until after delivery. We present a case of a large, symptomatic uterine fibroid diagnosed during pregnancy which was successfully managed by antepartum myomectomy.

**Case presentation:**

A 30 year old woman presented with a one year history of abdominal swelling, amenorrhea and severe epigastric discomfort of 19 weeks duration. The abdomen was grossly distended and tense. A sonographic diagnosis of ovarian tumor in pregnancy was made. Laparotomy revealed a 32 cm degenerating subserosal uterine fibroid co-existing with an intrauterine pregnancy. Myomectomy was successfully performed. The subsequent antenatal period was uneventful with a spontaneous vaginal delivery of a female baby at 38 weeks.

**Conclusion:**

This report supports other studies and case series that have demonstrated the safety of myomectomy during pregnancy in selected circumstances.

## Background

The prevalence of leiomyoma during pregnancy is reported as 2% [1]. During pregnancy, uterine leiomyoma are usually asymptomatic but may be occasionally complicated by red degeneration and an increased frequency of spontaneous abortion, preterm labor, premature rupture of fetal membranes, antepartum hemorrhage, malpresentations, obstructed labour, cesarean section and postpartum hemorrhage [1-3]. The management of uterine leiomyoma during pregnancy is largely expectant and its surgical removal is generally delayed until after delivery [4-7]. Because of the increased vascularisation of the uterus during pregnancy, women are at increased risk of bleeding and postoperative morbidity during myomectomy [2,5,6,8,9]. Some reports have shown that myomectomy during cesarean delivery can be safe [7,10-15]. Controversy persists among reports of myomectomy being performed during pregnancy [1], with some case series having reported the safety of antepartum myomectomy in carefully selected patients [1,16].

We present a case of a large symptomatic fibroid diagnosed during pregnancy which was successfully managed by antepartum myomectomy.

## Case Presentation

### History, examination and management

A 30-year old primigravida presented to our center on 17 October 2003 with a one year history of abdominal swelling and amenorrhea of 19 weeks duration. The abdominal swelling started as a small lump but markedly increased in size in the preceding 3 months. It was associated with pain, severe epigastric discomfort, constipation, weakness and swelling of the legs.

The patient was ill-looking, clinically pale and had bilateral pitting pedal edema. The pulse rate was 80 beats per minute and the blood pressure was 120/80 mmHg. The respiratory rate was 24 cycles per minute. The abdomen was grossly distended and tense. There was a massive central abdomino-pelvic mass which was firm and irregular, measuring 40 cm from the symphysis pubis.

Abdominal sonography showed an intra-uterine viable singleton fetus of 20 weeks gestation. It also showed a 30 cm multi-loculated cystic tumor with a thick capsule located at the right posterior-superior aspect of the uterus and free fluid in the peritoneal cavity. A sonographic diagnosis of ovarian tumor in pregnancy was made.

**Figure 1:**
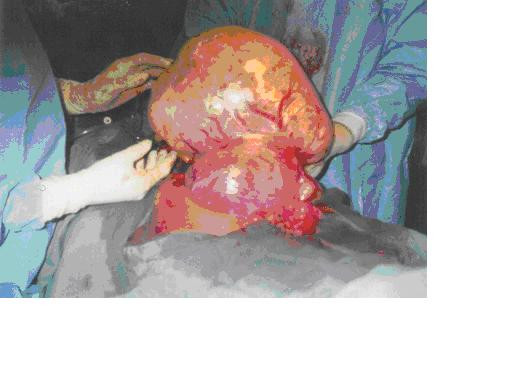
*Uterus with massive subserosal fibroid*. The photograph is JPEG format. It shows a cystic subserous fibroid measuring 32 cm in diameter and situated at the right posterior aspect of the uterus.

Blood tests showed a hematocrit of 22%, and normal electrolytes, urea and creatinin levels. The woman's blood group was 0 Rhesus positive and the hemoglobin genotype was AA. Malaria treatment was started following a positive smear test and two units of sedimented cells were administered to correct the anaemia. Because of the severity of the symptoms and the sonographic findings being suspicious of malignancy, surgery was proposed and discussed with the patient. Laparotomy was performed under general anaesthesia with endotracheal intubation. Operative findings included ascites, normal liver, spleen, kidneys, diaphragm, ovaries and fallopian tubes. The uterus was soft and the size was adequate for 20 weeks of gestation. Fetal movements were visible. A cystic subserosal fibroid measuring 32 cm in diameter was situated at the right posterior superior aspect of the uterus. [Fig 1]

The subserosal fibroid was adherent to the omentum and the anterior abdominal wall. It was removed and the myoma bed was quickly closed with 2-0 polyglactin suture and hemostasis was easily achieved. The estimated blood loss was 600 mls and 2 units of whole blood were transfused intra-operatively. The tumor weighing 7.7 kg was sent for histology.

Intravenous magnesium sulphate was administered to prevent uterine contractions and the woman had an uneventful post-operative follow up. The post-operative hematocrit was 30% and the woman was discharged from the hospital 10 days after the operation. The histology report showed sections of interlacing bundles of smooth muscles with areas of hyaline degeneration with no evidence of malignancy. Repeat sonography during antenatal care visits showed a normally growing fetus and the remainder of the antenatal period was uneventful. The woman went into spontaneous labor at 38 weeks gestation and delivered vaginally a female baby weighing 3.5 kg with Apgar scores of 8 and 10 at one and five minutes, respectively. Two days post partum the maternal hematocrit was 30% and mother and baby were discharged from the hospital. The 6 weeks post-natal visit was unremarkable.

## Discussion

To the best of our knowledge this is the first report of antepartum myomectomy from Nigeria. The decision to remove the fibroid was justified by its size and the patient's symptoms. The benefit was the relief of symptoms and a tissue diagnosis of a very large, suspicious abdominal mass. Its subserosal location may have contributed to easy enucleation and closure of the myoma bed. Hypercoagulability in pregnancy might have contributed to the ease in achieving hemostasis. The ease with which the fibroid was removed and the minimal measures used to obtain hemostasis contributed to the safety of the procedure. This case illustrates that myomectomy during pregnancy can be safely performed in carefully selected cases.

Antepartum myomectomy associated with reversal of fetal complications such as oligohydramnios, fetal postural deformity and intrauterine growth restriction has been reported [17].

This case also illustrates that cystic degeneration of a subserosal uterine fibroid is a differential diagnosis of ovarian tumor in pregnancy [18]. Sonography may be useful in evaluating the size, number, position, location, relationship to the placenta and echogenic structure [18] but it can be difficult to differentiate a complex ovarian mass from a degenerating fibroid.

## Conclusion

A degenerating uterine fibroid may mimic an ovarian tumor in pregnancy and obstetricians should be aware of the differential diagnosis. Although most cases of uterine fibroids in pregnancy can be managed conservatively, antepartum myomectomy may be necessary in selected cases.

## Competing interests

The author(s) declare that they have no competing interest.

## Authors' contributions

CU performed the surgery and conceived of the study. PFW did the literature search. Both authors collaborated in the preparation of the manuscript, read and approved the final manuscript.
